# A novel link between silent information regulator 1 and autophagy in cerebral ischemia-reperfusion

**DOI:** 10.3389/fnins.2022.1040182

**Published:** 2022-11-23

**Authors:** Yingying Tang, Jiaqian Xie, Xiaoping Chen, Lihong Sun, Lili Xu, Xinzhong Chen

**Affiliations:** Department of Anesthesiology, Women’s Hospital, School of Medicine, Zhejiang University, Hangzhou, China

**Keywords:** cerebral ischemia-reperfusion, SIRT1, neuroprotection, autophagy, autophagy signaling pathway

## Abstract

Cerebral ischemia is one of the leading causes of death and disability worldwide. Although revascularization *via* reperfusion combined with advanced anticoagulant therapy is currently a gold standard treatment for patients, the reperfusion itself also results in a serious dysfunction termed cerebral ischemia-reperfusion (I/R) injury. Silent information regulator 1 (sirtuin 1, SIRT1), is a classic NAD^+^-dependent deacetylase, which has been proposed as an important mediator in the alleviation of cerebral ischemia through modulating multiple physiological processes, including apoptosis, inflammation, DNA repair, oxidative stress, and autophagy. Recent growing evidence suggests that SIRT1-mediated autophagy plays a key role in the pathophysiological process of cerebral I/R injury. SIRT1 could both activate and inhibit the autophagy process by mediating different autophagy pathways, such as the SIRT1-FOXOs pathway, SIRT1-AMPK pathway, and SIRT1-p53 pathway. However, the autophagic roles of SIRT1 in cerebral I/R injury have not been systematically summarized. Here, in this review, we will first introduce the molecular mechanisms and effects of SIRT1 in cerebral ischemia and I/R injury. Next, we will discuss the involvement of autophagy in the pathogenesis of cerebral I/R injury. Finally, we will summarize the latest advances in the interaction between SIRT1 and autophagy in cerebral I/R injury. A good understanding of these relationships would serve to consolidate a framework of mechanisms underlying SIRT1’s neuroprotective effects and provides evidence for the development of drugs targeting SIRT1.

## Introduction

Cerebral ischemia is a common form of ischemic stroke caused by a decrease in blood flow to the brain. According to statistics from the World Health Organization, about 5 million deaths and 5 million permanently disabled are caused by ischemic stroke, bringing a heavy economic burden on families and society (Singh et al., 2020). Currently, clinical treatment is mainly thrombolysis. However, the time window of intravenous thrombolysis is very short, only 3–8% of patients can be rescued. Additionally, giving medication outside of the therapeutic window would increase the risk of intracranial hemorrhage (ICH), which can further exacerbate tissue damage. Moreover, a secondary injury caused by reperfusion known as ischemic–reperfusion (I/R) injury can induce excessive oxidative stress, calcium overload, cell apoptosis and neuronal damage (Albers et al., 2018; Nogueira et al., 2018; Thomalla et al., 2018; Jung et al., 2021). Therefore, it is urgent to find more suitable and clinically actionable treatment for this major medical problem. In the last decade, numerous molecular targets have been found by researchers to control ischemia cell death. Growing evidence suggests that Sirtuins, and particularly SIRT1, play a crucial role in neuroprotection against ischemic stimuli.

Sirtuins are highly evolutionarily conserved proteins which require oxidized nicotinamide adenine dinucleotide (NAD^+^) as a cofactor for their enzymatic action. Sirtuins are expressed throughout the body, including the heart, muscle, liver, kidney and brain. In response to cellular stress, they coordinate the regulation of numerous biological processes and pathways, including the cell cycle, DNA repair, cell survival, aging, mitochondria biogenesis and autophagy. In mammalian cells, the Sirtuin family consists of seven members sirtuin1-7 (SIRT1-7), among which SIRT1 is the most characterized and studied. SIRT1 is the mammalian homologue of yeast SIRT2 and has multiple regulators and targets. Histone and non-histone are significant targets of SIRT1. Through targeting of histone and non-histone proteins as well as downstream specific protein substrates, it has been a crucial mediator in various biological processes including cell survival, oxidative stress, inflammation and autophagy (Kitada et al., 2016).

Autophagy is a natural, regulated process that eliminates dysfunctional organelles and proteins to maintain tissue homeostasis. Autophagy can be initiated during cerebral ischemia when the neuronal cells are subjected to the risk of nutrients and oxygen insufficiency. Notably, autophagy is a “double-edged sword” in the pathophysiology of cerebral I/R injury. Although there is controversy on the role of autophagy in cerebral ischemia, it is universally believed that moderate autophagy is beneficial while excessive autophagy is harmful to brain cells (Mo et al., 2020). The efficacy and mechanisms of autophagy regulation in cerebral I/R injury need to be further clarified. Recently, with further research into autophagy being studied, SIRT1 has also been found to regulate autophagy in multiple human diseases through activating different signaling pathways, such as the SIRT1-FOXO3 pathway, AMP-activated protein kinase (AMPK) pathway and p53 pathway. Interestingly, recent growing evidence suggests that the neuroprotective effects of SIRT1 are associated with autophagy regulation in the pathophysiological process of cerebral ischemia. Thus, in our review, a comprehensive overview of the specific roles of autophagy in cerebral I/R injury will be discussed. At the same time, the possible protective effects of SIRT1 through autophagy modulation during cerebral I/R injury will be described in detail.

## Role of silent information regulator 1 in cerebral ischemia-reperfusion injury

### Beneficial role of silent information regulator 1 in cerebral ischemia-reperfusion injury

Silent information regulation protein 1 deacetylates a wide range of histone and non-histone substrates to regulate gene expression and participates in numerous physiological processes (Zhang et al., 2018). Previous multiple studies considered SIRT1 as a pro-longevity regulator against aging-related diseases like neurodegeneration and cardiovascular disease (Donmez, 2012; Chen et al., 2020b). In recent years, SIRT1 has been reported to have protective effects against cerebral I/R injury in experimental studies of diverse ischemic stroke models, including focal, or global cerebral ischemia (GCI). In SIRT1-deficient mice, ischemia-perfusion injury is worsened by increased levels of inflammation, oxidative stress, and apoptosis, whereas activation of SIRT1 alleviates ischemic injury (Wang et al., 2016). Here, we summarized molecular mechanisms and new findings of SIRT1 in different cerebral ischemia conditions below.

### Silent information regulator 1 in focal cerebral ischemia (FCI)

A growing body of evidence indicated that the neuroprotection of SIRT1 was associated with the regulation of multiple molecular signaling pathways. It was reported that SIRT1 regulated mitochondrial function and antioxidant defenses through activation of transcriptional coactivator PPAR-γ co-activator1α (PGC-1α) and activities and the anti-oxidant enzyme SOD after brain ischemic damage in rats subjected to transient middle cerebral artery occlusion (tMCAO), a classic FCI animal model (Li et al., 2021). SIRT1 could inhibit oxidative stress in the tMCAO rats by activation of Nrf2, NQO1, and HO-1, which are important factors mediating oxidative stress response (Mei et al., 2022). Besides, the SIRT1/FOXO1 pathway was also reported to be activated by SIRT1 to alleviate excess oxidative injury. Deacetylated FOXO1 increased the clearance of reactive oxygen species (ROS), thereby inhibiting excessive oxidative stress in C57BL/6 mice of transient FCI (Wang et al., 2020b). SIRT1/VEGF pathway was demonstrated to promote angiogenesis by increasing microvascular density in the boundary ischemic area after cerebral I/R injury in MCAO-induced Sprag-Dawley rats (Zheng et al., 2018). Moreover, the NAD^+^/SIRT1 signaling pathway also was reported to regulate ischemia-induced blood-brain barrier disruption in ischemic mice (Zhang et al., 2022a).

Apart from regulating signaling pathways, SIRT1 could exert neuroprotective effects *via* the regulation of microRNAs, such as miR-22. Through modulating miR-22 expression, SIRT1 inhibits the activation of the Bcl2-associated X (Bax) signaling pathway and caspase-3 activity, reduces inducible cyclooxygenase (COX)-2 and nitric oxide synthase (iNOS) levels, decreases interleukin 6 (IL-6) and tumor necrosis factor-α (TNF-α) activity in lipopolysaccharide-induced tMCAO rats, thereby reducing apoptosis and neuroinflammation (Lu and Wang, 2017).

### Silent information regulator 1 in global cerebral ischemia

Overexpression of SIRT1 was observed in microglial cells under the NAD^+^ treatment and alleviated mitochondrial damage and ROS production in microglia, thus improving cognitive function and reducing neuroinflammation in chronic brain hypoperfusion rats within bilateral common carotid artery occlusion (BCCAO), a common animal model for GCI. The study further denoted that the SIRT1’s neuroprotective properties was associated with the activation of the SIRT1/PGC-1α signaling pathway (Zhao et al., 2021). Similarly, another study showed that the expression of SIRT1 and PGC-1α upregulated under the treatment of Coenzyme Q10 (CoQ10) and near-infrared (NIR) photobiomodulation (PBM) in mice of transient GCI. The researchers further demonstrated that activation of the SIRT1/PGC1-α/NRF1/TFAM signaling pathway could modulate neuronal mitochondrial biogenesis, and decrease delayed cell death in aged ischemic brains (Salehpour et al., 2019).

Additionally, another investigation found that mild hypothermia increased the survival rate and ameliorated apoptosis in the cortex of mice after cardiac arrest/cardiopulmonary resuscitation (CA/CPR) through SIRT1-mediated autophagy, whereas inhibition of SIRT1 abolished the neuroprotection of hypothermia both *in vivo* and *in vitro* (Wei et al., 2019). Similarly, an *in vivo* study of GCI revealed that stimulation of SIRT1/FOXO1-mediated autophagy after reperfusion under hypoxic postconditioning alleviated GCI-induced cognitive damage and improved neurological outcomes in ischemic rats (Liu et al., 2021). Moreover, a recent study focusing on the intervention of delayed cerebral ischemia (DCI) in aneurysmal subarachnoid hemorrhage (SAH) demonstrated that hypoxic postconditioning after SAH provides significant resilience against cerebral vasospasm, microvessel thrombi, and neurological impairments in a SIRT1-dependent manner, whereas SIRT1knockout mice (SIRT1^–/–^) are more susceptible to SAH-induced DCI (Diwan et al., 2021).

### Silent information regulator 1 activators for the treatment of cerebral ischemia-reperfusion injury

Accumulating studies have revealed that some small chemicals and molecules can activate SIRT1, making it a potentially effective therapeutic target for the treatment of cerebral I/R injury (Wang et al., 2020a; Shi et al., 2021a). Resveratrol, as the first SIRT1 activator, has been reported to have neuroprotective benefits in cerebral ischemic injury (Meng et al., 2015; He et al., 2017a; Grewal et al., 2019; Yu et al., 2021). In addition, SRT2104, a novel SIRT1 activator, has also been observed to inhibit neuron and microglia death and regulate microglia polarization *via* the SIRT1/NF-κB axis in the model of oxygen-glucose deprivation/reoxygenation (OGD/R), a model of hypoxia/ischemia (Fu et al., 2021).

Apart from small molecules, several pharmacological compounds have been shown to exert neuroprotective effects against ischemic stroke by activation of SIRT1 with various mechanisms, including antiapoptotic, anti-inflammatory and antioxidative effects. For example, maresin 1 attenuated inflammation and mitochondrial damage in MCAO mice *via* stimulating SIRT1 signaling (Xian et al., 2019). Cycloastragenol isolated from Astragalus Radix was found to provide a neuroprotective effect against ischemic brain injury in tMCAO-induced ischemic mice, reducing the ratio of Bax/Bcl-2 and suppressing the mRNA expression of pro-inflammatory cytokines through the upregulation of SIRT1 (Li et al., 2020). Astragaloside IV was reported to improve the neurological deficit and reduced the cerebral infarction area by the SIRT1/Mapt pathway in MCAO rats (Shi et al., 2021b). Oxymatrine attenuated histological injury and cognitive deficits, inhibited apoptosis and promoted autophagy by elevating SIRT1 levels in BCCAO-induced cerebral I/R rats (Zhou et al., 2018). However, given that the bioavailability and efficacy of these compounds in humans are not well-known, thus the effectiveness of these drugs for the treatment of diseases, where SIRT1 levels are regulated, remains to be further determined.

### Potentially detrimental role of silent information regulator 1 in cerebral ischemia-reperfusion injury

As is commonly known, neurons are vulnerable to excitotoxicity due to high energy needs for cell survival and function. ATP is a major source of energy for neurons, which is generated during mitochondrial oxidative phosphorylation. NAD^+^ functions as a source of high-energy phosphate and an adenosine donor for the synthesis of ATP. Maintenance of NAD^+^ and the NAD^+^ /NADH ratio is crucial to cell survival both in healthy physiological and pathological stress such as ischemia (Ying and Xiong, 2010). Notably, significant reductions in NAD^+^ (and alteration of its relative NADH) after ischemia are associated with energy depletion which induces cell death. Preservation of NAD^+^ is vital for improved outcomes, evidenced by the research that replenishing cellular NAD^+^ both before and after OGD dramatically alleviates ischemia damage (Wang et al., 2008).

Since SIRT1 requires NAD^+^ for enzymatic activity, it may deplete cellular energy and possibly make neurons more susceptible to ischemia and excitotoxicity. An *in vitro* study demonstrated that nicotinamide (a SIRT1 inhibitor) protects neurons against excitotoxicity and prevents NAD^+^ depletion, whereas SIRT1 may impair energetically compromised neurons by consuming NAD^+^ in cerebral ischemia (Liu et al., 2009). Similarly, another *in vivo* study reported that SIRT1 overexpression in mice alleviates memory deficit, but not exerts neuroprotection while excess SIRT1 might consume NAD^+^ necessary for neuron survival (Kakefuda et al., 2009). Further research is required to elucidate the effects of SIRT1 activity on NAD^+^ depletion in the setting of ischemia.

## Role of autophagy in cerebral ischemia-reperfusion injury

### An overview of autophagy in cerebral ischemia-reperfusion injury

Autophagy is a strictly controlled, regulated process that aids cells in getting rid of defective components, such as damaged organelles, long-lived proteins and degraded proteins to maintain tissue homeostasis (Chen et al., 2020a; Mizushima and Levine, 2020; Yang and Klionsky, 2020). According to the manner of cargo delivery to the lysosomes, autophagy is proposed to be subdivided into three types: macroautophagy, microautophagy and chaperone-mediated autophagy (CMA). Macroautophagy is the most prevalent form of autophagy, including the following four steps: (a) initiation of autophagy, (b) vesicle nucleation (c) vesicle elongation, and (d) fusion and degradation. Through the aforementioned processes, autophagy accomplishes the metabolic and renewal demands of cells, recycling the denatured organelles and misfolded proteins for reuse to maintain cellular homeostasis (Levine and Kroemer, 2019; Ajoolabady et al., 2021). Autophagy has been suggested to be involved in the development of various diseases, such as stroke (Thiebaut et al., 2019), cancer (Amaravadi et al., 2019; Mariotto et al., 2020), neurodegenerative disease (Tran and Reddy, 2021), cardiovascular disease (Del Re et al., 2019; Phadwal et al., 2020), and so on.

Interestingly, recent numerous studies have shown that autophagy participates in the development of cerebral ischemia and is thought of playing a dual role in cerebral I/R injury. Some *in vivo* and *in vitro* studies found that autophagy is deleterious to the outcomes of cerebral I/R injury (Baek et al., 2014; Feng et al., 2017; Liu et al., 2019). However, additional studies revealed that autophagy is neuroprotective for brain damage (Tripathi et al., 2016; Hwang et al., 2017). These research’ contradictory findings could be the result of using various cerebral I/R damage models and dosing agents, which would affect the timing and degree of autophagy regulation. Of note, emerging evidence indicates that sex differences also play a significant role in autophagy regulation through gonadal hormones receptors and autophagic pathway-related genes located on the X chromosome, resulting in the different outcomes within different genders (Azcoitia et al., 2019; Shang et al., 2021).

Although the specific role of autophagy in cerebral I/R injury is controversial, it is unanimously believed that mild to moderate activation of autophagy at the early stages of cerebral I/R injury may have a pro-survival effect while excessive or persistent activation of autophagy may have a pro-death effect during cerebral I/R injury.

### Autophagy pathways in cerebral ischemia-reperfusion injury

Recent investigations have revealed multiple molecular mechanisms of autophagy involved in cerebral I/R injury. It was reported that the AMPK/mTOR1/ULK1 pathway played a crucial role in mediating autophagy during GCI (Lai et al., 2020). I/R damage exposes neuronal cells to nutrient-depleted conditions, which increases the AMP/ATP ratio and serves as a primary trigger for the activation of the AMPK pathway (Jiang et al., 2018). AMPK directly phosphorylates ULK1 at several serine residues, inducing autophagy and also inhibiting mTORC1 activity and indirectly reducing mTOR-evoked phosphorylation of ULK1, leading to enhanced interaction between AMPK and ULK1 (Kim et al., 2011). In addition, the regulation of autophagy by AMPK can be blocked by phosphorylation of different subunits of the same class III PI3K complex, AMPK activation both enhances the phagocytic vps34 complex and inhibits other autophagy-independent class III PI3K complexes formation (Tamargo-Gomez and Marino, 2018). p38 and JNK play an critical role in inflammatory-mediated ischemic damage (Sun and Nan, 2016). p38α-MAPK pathway plays a crucial role in alleviating inflammation by regulation of autophagy. It was found that in lipopolysaccharide (lipopolysaccharide, LPS) stimulated microglia, p38α MAPK can directly phosphorylates and inhibits ULK1 in the autophagy initiation complex, disrupting the ATG1 interaction in the autophagy, thereby reducing the flux and level of autophagy, suggesting that activation of the p38α-MAPK pathway can inhibit the level of autophagy (He et al., 2018). However, in several other studies, the regulation of autophagy by p38-MAPK pathway was reversed. Xue et al. (2016) found that inhibiting the JNK/p38 cascade pathway negatively regulated autophagy levels in OGD-treated PC12 cells, and inhibited the activation of apoptosis. Zhang et al. (2015) found that inhibition of the p38-MAPK pathway reduced mitochondrial filamentation and mitophagy, which maintains mitochondrial content in ischemic stroke. Additionally, Luo et al. (2022) found that BNIP3/LC3 pathway upregulated autophagy and inhibited NLRP3 inflammasome-derived inflammatory response during cerebral I/R injury. Apart from the abovementioned mechanisms, autophagy was also thought to be modulated by the AKT1-GSK-3β axis, the FKBP5-AKT1-FOXO3 mediated-maladaptive autophagy, FOXOs pathway, PINK1/Parkin pathway, mTOR pathway, and so on (Guo et al., 2018; Sun et al., 2019b; Yu et al., 2020).

## Silent information regulator 1 and autophagy pathways in cerebral ischemia-reperfusion injury

Numerous transcriptional and signaling pathways have been identified in the modulation of autophagy in studies on cerebral ischemia. As previously mentioned, SIRT1 catalyzes the deacetylation of histones H1, H3, and H4 as well as non-histone substrates, including HIF-1a, Forkhead box O (FOXOs), p53 and AMPK, which are known to regulate autophagy. SIRT1 was first reported to regulate autophagy by directly deacetylating autophagy genes (Atg)5, (Atg)7, and (Atg)8 in a NAD-dependent manner (Lee et al., 2008). Recently, accumulating evidence has revealed that SIRT1 participate in autophagy regulation in cerebral I/R injury *via* the FOXOs pathway, AMPK pathway, PINK1/Parkin pathway, mTOR pathway and p53 signal pathway (Table 1). Here, the novel associations between SIRT1 and autophagic signaling pathways during I/R injury will be discussed below.

**TABLE 1:** Targets of silent information regulator 1 (SIRT1) that are modulated during the autophagy process.

Substrates	Models	Functions of SIRT1	Mechanism	References
FOXO1	MCAO rats	ameliorated autophagy and protected the neurons	deacetylated FOXO1 and further reduced interaction with Atg7	Mei et al., 2020
FOXO3	HT22 cells of I/R model	inhibiting autophagy and oxidative stress response	deacetylated FOXO3a and increased signal pathway	Wu et al., 2020
PINK1/Parkin	OGD/R-induced HT22 cells	enhanced mitophagy and alleviated neuronal apoptosis	increased PINK1 levels and the transfer of Parkin to mitochondria	Shao et al., 2021
AMPK	neuroblastoma N2a cells	modulates autophagic degradation in neuronal cells	deacetylated LKB1 and subsequent phosphorylated AMPK	Park et al., 2016
mTOR	transient cerebral I/R aged rats	induced autophagy and alleviated neuronal damage	reduced the phosphorylation of mTOR	Wang et al., 2019
p53	cerebral ischemia pup rats	increased autophagy activation and reduced intrinsic apoptosis	reduced the acetylated p53 levels	Carloni et al., 2017

### Forkhead box O

Forkhead box O transcription factor family, a class of key homeostasis regulators, consists of four members FOXO1, FOXO3, FOXO4, and FOXO6 in mammalian cells (Calissi et al., 2021). It has been shown that the FOXO family plays a pivotal role in various cellular processes, including oxidative stress, apoptosis, stress resistance, immune response, autophagy, and inflammation responses. The transcriptional activation ability of FOXOs is tightly regulated by a range of significant post-translational modifications such as acetylation/deacetylation, phosphorylation/dephosphorylation and ubiquitination (Cheng, 2019). It has been demonstrated that the FOXO family function as a SIRT1 deacetylates member that influences downstream autophagy-related pathways and regulates autophagy in numerous pathophysiological processes of diabetes, obesity, cardiovascular disease, tumor, aging, and other diseases (Zhu et al., 2019; Mao et al., 2021; Rong et al., 2021).

FOXO1 is an essential autophagy mediator in diverse organs, including the heart, skeletal muscle, and brain. It has been reported to be deacetylated by SIRT1 in cerebral I/R injury rats, and decreased acetylated FOXO1 levels in the cytosol further reduced the interaction with autophagy-related gene 7 (Atg7), one of the key molecules in autophagy, thus alleviating excessive autophagy and neurological deficits in cerebral ischemic rats (Mei et al., 2020). Additionally, another study reported that hypoxic postconditioning elevated the levels of the autophagy-related protein LC3-II in the hippocampus and exerted protective effects *via* the SIRT1/FOXO1-dependent pathway in rats of GCI (Liu et al., 2021).

FOXO3a is another important member of the FOXO family and also is an important downstream molecule of SIRT1. SIRT1 deacetylates and enhances the capacity of FOXO3a to trigger the production of antioxidant proteins such as superoxide dismutase (SOD), thus optimizing cell survival against oxidative stress (Wang et al., 2017). Recent emerging evidence has shown that FOXO3a was involved in regulating mitophagy through controlling transcription of downstream ubiquitous autophagy-related genes, including PINK1, sequestosome, and BNIP3 (Song et al., 2017; Jiang et al., 2022). BNIP3 is a crucial mitochondrial autophagy receptor, and FOXO3 controls mitochondrial activity and integrity by attaching to the BNIP3 upstream promoter region (Lu et al., 2014). SIRT1 silencing alone is sufficient to enhance FOXO3 acetylation, decreasing the binding of BNIP3 and FOXO3, thus inhibiting BNIP3 transcription in hypoxia-exposed aged kidney mice (Kume et al., 2010). In addition, a study of hepatocellular carcinoma demonstrated that CDK9 inhibition reduced SIRT1 phosphorylation and SIRT1-mediated deacetylation of FOXO3, and inhibited FOXO3 activity and BNIP3 transcription, thereby blocking the activation of PINK1-PRKN-mediated mitophagy (Yao et al., 2021). Furthermore, a recent study of cerebral I/R injury reported that LncRNA SNHG12 reduced oxidative stress and improved cell activity by inhibition of SIRT1/FOXO3a-mediated autophagy in HT22 cells and overexpression of cells co-transfected with SIRT1 and FOXO3 further decreased the levels of MDA and ROS (Wu et al., 2020). Interestingly, an investigation focusing on renal injury revealed that Cordyceps cicadae ameliorated renal fibrosis and delayed hypertensive nephropathy progression through SIRT1/FOXO3a/ROS signaling-mediated autophagy in spontaneously hypertensive rats and the NRK-52E cells. Moreover, in this study, upregulation of SIRT1/FOXO3a increased the levels of SOD and decreased the levels of MDA, both of which regulate the production of ROS and alleviated oxidative stress that led to autophagic stress (Cai et al., 2021). As is common knowledge, there are two stages in cerebral I/R injury: the ischemic phase and the reperfusion phase. During the reperfusion phase, a significant amount of ROS production was thought to trigger autophagic stress and oxidative stress rapidly. Therefore, we propose that when cerebral reperfusion occurred, SIRT1 may inhibit an excessive autophagy process by removing ROS. There might be a crosstalk biological mechanism for modulating autophagic activity through activating multiple SIRT1-mediated downstream signaling pathways in the reperfusion phase. Whereas more investigation is needed to determine the precise mechanisms involved in the different I/R phases.

### PTEN-induced putative kinase 1/Parkin RBR E3 ubiquitin-protein ligase

Mitochondria are vital organelles in eukaryotic cells, regulating a series of complex physiological processes, such as calcium signaling, redox homeostasis, and cellular energy metabolism (Chan, 2020). As mitochondrial dysfunction often brings serious consequences for intracellular homeostasis and accelerates tissue damage, timely clearance of dysfunctional mitochondria is particularly crucial to maintain a healthy mitochondrial network for cellular homeostasis (Mu et al., 2020). The concept of “mitophagy” was firstly proposed by Lemasters (2005) who demonstrated that mitophagy selectively removed the impaired mitochondria in cells, which is beneficial to mitochondrial homeostasis and cell survival. Multiple lines of evidence indicated that moderately increased mitophagy is considered to be adaptive or protective for neurons in cerebral I/R injury.

PTEN-induced putative kinase 1 (PINK1) and Parkin RBR E3 ubiquitin-protein ligase (Parkin) are two pivotal mitophagy regulating factors in mammalian cells. Under normal cellular conditions, PINK1 is constitutively imported to the mitochondrial inner membrane. When I/R injury occurs, mitochondrial depolarization inhibits the degradation of PINK1. Subsequently, PINK1, accumulating in the depolarized mitochondrial outer membrane, recruits Parkin to the damaged mitochondria and directly activates Parkin E3 ligase activity by phosphorylation and ubiquitin. After Parkin activation, mitophagy is induced by ubiquitinated mitochondrial proteins and mitophagy receptors. PINK1/Parkin-mediated pathway has been considered the most well-characterized mitophagy signaling pathway, which could also be modulated by diverse molecular mechanisms (Tanaka, 2020). Recent studies have shown that SIRT1 could regulate PINK1/Parkin-dependent mitophagy (Liu et al., 2020; Yi et al., 2020). In an *in vitro* study of diabetic lung I/R injury, activation of SIRT1-PINK1-dependent mitophagy reduced the apoptotic index, serum concentrations of TNF-a and IL-6, MDA and mitochondrial ROS production, thus alleviating reperfusion-induced cell apoptosis, oxidative stress and mitochondrial dysfunction (Jiang et al., 2021). In addition, in HT22 cells of the OGD/R model, Apelin-36 treatment activated SIRT1, subsequently promoting PINK1 expression levels consistent with the translocation of Parkin to mitochondria and alleviating mitochondrial apoptosis and improving cell viability (Shao et al., 2021). Furthermore, enhanced stability of SIRT1 by overexpression of Ceramide Kinase-Like Protein (CERKL) promoted the production of PINK1 and Parkin in human neuroblastoma cells of OGD/R. CERKL alleviated neuronal damage through activating mitophagy in a SIRT1/PINK1/Parkin-dependent pathway (Huang et al., 2022). Thus, proper modulation of PINK1/Parkin-mediated mitophagy by activating SIRT1 may be beneficial in the treatment of I/R injury.

### Adenosine monophosphate-activated protein kinase

Adenosine monophosphate-activated protein kinase (AMPK) is an evolutionarily conserved serine/threonine protein kinase, which plays a key role in cell survival and cellular energy balance (Jeon, 2016). Apart from regulating a wide range of metabolic functions, AMPK is also one of the most conserved autophagy inducers and its activity is related to autophagic degradation in almost all eukaryotic cells (de Pablos et al., 2019; Saikia and Joseph, 2021).

Studies have revealed a strong relationship between SIRT1 and AMPK in autophagy regulation since they can reciprocally influence each other’s activity in distinct ways (Ganesan et al., 2017; Li et al., 2021). On one hand, as an important cellular energy sensor, the activity of AMPK is tightly controlled by multiple upstream regulators. Liver kinase B1 (LKB1) is one of the primary upstream regulators of AMPK, it has been reported that SIRT1 can deacetylate LKB1 at Lys48 and activate LKB1, which increases phosphorylation of AMPK at Thr172 (Lan et al., 2008). The SIRT1/LKB1/AMPK pathway has a vital role in attenuating Tau phosphorylation and Aβ accumulation in neuronal cells (Park et al., 2016). Additionally, an investigation revealed that melatonin treatment increased SIRT1 activity and p-AMPK levels, enhanced the autophagy activity of nerve cells and promoted recovery of motor function following spinal cord injury (SCI) (Gao et al., 2020).

On the other hand, AMPK activation elevated cellular NAD^+^ levels, leading to SIRT1 activation and deacetylation of targets such as PGC1α and FOXOs. A recent study revealed that activation of AMPK *via* ghrelin treatment promoted mitophagy and improved cell viability, and mitochondrial biogenesis and stimulated SIRT1-PGC1α signaling in SH-SY5Y cells after rotenone-induced cytotoxicity (Wang et al., 2021). In addition, an investigation focusing on subarachnoid hemorrhage revealed that resveratrol significantly decreased the activation of microglia and the release of inflammatory cytokines, which alleviated brain edema and neurological behavior impairment *via* the AMPK/SIRT1-mediated autophagy signaling pathway (Li and Han, 2018). Interestingly, another study described a novel AMPK-GAPDH-SIRT1 pathway in a glucose starvation model, in which AMPK phosphorylated GAPDH and caused nuclear translocation of GAPDH, then nucleus GAPDH interacted directly with SIRT1, replacing SIRT1’s repressor, leading to SIRT1 activation and autophagy initiation. This study suggested that Sirt1 participates in AMPK-evoked rapid post-translational processes where SIRT1 activation by AMPK interacts directly with GAPDH and is independent of NAD^+^ up-regulation (Chang et al., 2015). Moreover, resveratrol was reported to increase the phosphorylation of AMPK in the cerebral cortex and decrease the infarct area in MCAO rats. It also promoted mitophagy (lower levels of LAMP1 and the mitochondrial matrix protein HSP60, favored the recruitment of LC3-II and increased Beclin1 levels) in neuronal cultures exposed to glutamate-induced excitotoxicity (Pineda-Ramírez et al., 2020). Therefore, pharmaceutical manipulation of SIRT1 and AMPK proteins may offer a potentially useful strategy to reduce increased vulnerability to ischemia insults.

### Mammalian target of rapamycin

Mammalian target of rapamycin (mTOR) is a master regulator during multiple physiological processes including cell growth, metabolism and differentiation. mTOR is also essential for regulating autophagy in a variety of diseases (Kim and Guan, 2015).

Accumulating data have shown that the SIRT1/mTOR pathway is a critical autophagy-related signaling cascade in response to cellular stress. SIRT1 reversed the autophagy impairment caused by ischemic insults and alleviated ischemic damage *via* inhibiting the mTOR pathway (Wang et al., 2019). Additionally, it has been observed that SIRT1 inhibition activates the mTOR signal pathway, which results in autophagy damage (Tae et al., 2020). Increased expression of SIRT1 during 17β-estradiol enhanced the expression of p-AMPK and mitophagy-related proteins, but decreased the expression of p-mTOR, promoted mitophagy activation and improved cell viability and proliferation in mouse chondrocytes (Mei et al., 2021). Nicotinamide phosphoribosyltransferase (Nampt), the rate-limiting enzyme in mammalian NAD^+^ biosynthesis, increased autophagy and improved neuronal survival by modulating the TSC2-mTOR-S6K1 signaling pathway in a Sirt1-dependent manner following cerebral ischemic injury (Wang et al., 2012). Additionally, Activation of SIRT1 by urolithin A supplementation downregulated the mTOR signaling pathway, ameliorated apoptosis and rescued impaired autophagy in brain aging mice (Chen et al., 2019). Furthermore, SIRT1 activator resveratrol has been demonstrated to exert neuroprotection *via* PI3K/AKT/mTOR pathway and attenuated cerebral infarct volume and neuronal apoptosis in MCAO rats (Hou et al., 2018). Similarly, an investigation focusing on transient cerebral I/R injury revealed that enhanced expression of SIRT1 by NAD^+^ biosynthesis-related essential enzyme suppressed the relative phosphorylation of mTOR (pmTOR/mTOR) levels in the peri-infarct penumbra, regulated autophagy and alleviated ischemia-induced cortical neurons death in aged ischemic rats (Wang et al., 2019).

### p53

p53 is a crucial tumor suppressor which is commonly mutated in human cancer but is also an important transcription factor induced by cellular stress, which can regulate cell cycle arrest, apoptosis, DNA repair, differentiation, and autophagy (Levine and Oren, 2009).

Recently, emerging evidence indicated that there is a reciprocal functional interaction between autophagy and p53. Autophagy suppresses p53 by oxidative or other stress pathways and also p53 regulates autophagy by a suite of autophagy-related target genes (White, 2016). SIRT1 is known to directly deacetylate p53 at the C-terminal Lys382 residue and regulate the function of p53. A growing body of research has shown that SIRT1-mediated p53 signaling participates in the regulation of autophagy of ischemic diseases. A recent investigation focusing on acute kidney injury showed that pharmacologic activation of SIRT1 attenuated pathological renal damage *via* reducing the acetylation level of p53 at lysine 379 and promoting autophagy, ultimately prolonging the survival times of septic mice (Sun et al., 2021). In addition, another study found that upregulation of SIRT1 with apigenin treatment ameliorated inflammatory responses and oxidative stress injury by stimulating the SIRT1-p53 mediated-autophagy axis in Acetaminophen (APAP)-induced liver injury mice (Zhao et al., 2020). An experimental investigation of cerebral hypoxia-ischemia indicated that increased activity of SIRT1 by melatonin diminished activation of the early stages of intrinsic apoptosis, outer mitochondrial membrane permeabilization and glial cells activation in the neonatal rat brain through the SIRT1-p53-mediated signaling autophagy (Carloni et al., 2017). Similarly, simvastatin preconditioning preserved SIRT1 expression, suppressed p53 expression and acetylation levels and conferred neuroprotection against hypoxia ischemia-induced brain damage in hypoxic-ischemic neonatal rats (Carloni and Balduini, 2020). Furthermore, a recent study of OGD/R injury found that enhanced expression of SIRT1 by protocatechualdehyde treatment inhibited the expression of P53 and cell apoptosis, promoted autophagy (the expression level of autophagy markers P62, LC3 II/I and Beclin-1 increased), and ultimately significantly increased cell proliferation, cell survival rate and antioxidant activity in OGD/R-induced endothelial cells injury (Cao et al., 2022). Thus, p53 activity inhibition by SIRT1 may be a promising therapeutic approach for the management of I/R injury.

## Silent information regulator 1 and autophagy in ischemic astrocytes, microglial and endothelial cells

### Astrocytes

In the detrimental secondary process of cerebral I/R injury, diverse cell types of the central nervous system (CNS), including neurons, glial cells, vascular endothelial cells and other cell types, all interact to collectively affect the occurrence, development, and outcomes of cerebral ischemic responses. Among these cells, astrocytes are the most abundant glial cells in the CNS, which are essential in maintaining brain homeostasis, synapse formation, and nutrition of the CNS. After cerebral I/R injury, astrocytes are activated by multiple damage-associated molecular patterns (DAMPs) and cytokines and play a dual role in the complex cascade of ischemic stroke (Shen et al., 2021).

A recent study focusing on traumatic brain injury (TBI) showed that the SIRT1/MAPKs axis conferred neuroprotection against TBI through inhibition of overactivated astrocytes in interleukin-1β (IL-1β)-induced primary cortical astrocyte model and a murine model of nigrostriatal pathway damage. Suppression of astrocyte activation reduced the mean neurological severity score (mNSS) and improved the neurobehavioral function in brain injury mice (Li et al., 2017). To clarify the underlying mechanisms of SIRT1 alleviating astrocyte activation, the subsequent investigation was undertaken and the results demonstrated that decreased astrocyte activation (regulated by SIRT1) is related to the activation of astrocyte autophagy in the animal model of TBI. The study found that upregulation of SIRT1 by resveratrol increased the LC3 levels around the lesion site. Moreover, SIRT1 overexpression enhanced the LC3 levels in astrocytes but reduced the expression in the neurons, while nearly no effect was observed on the microglial cells. Mechanically, regulation of SIRT1 expression enhanced astrocytes autophagy around the lesion site and decreased neurons autophagy in the striatum, which may be associated with the distribution of LC3 in the nucleus and cytoplasm by measuring subcellular localization of LC3 with a confocal fluorescence microscope (Zhang et al., 2019). Interestingly, the researchers further found that the mechanism of reduced astrocyte activation by SIRT1 after brain damage was associated with regulating chaperone-mediated autophagy (CMA) in close head injury (CHI) (Zhang et al., 2022b). SIRT1 overexpression improved neurological functions, reduced astrocyte activation and rescued neuron survival after CHI, which was aborted by DnaJ heat shock protein family member B1 (Dnajb1)-shRNA administration. The study indicated that SIRT1 enhanced CMA activity by modulating the deacetylation and ubiquitination of Dnajb1during the process of CHI.

### Microglial cells

Microglia are central innate immune cells and highly plastic cells in the CNS, which are responsible for immune surveillance, brain homeostasis, neurogenesis and angiogenesis. After brain ischemia, microglial cells are activated by various damage-related molecular pattern molecules, molecular pathways and mediators, and serve a dual role in different pathological phases of cerebral I/R injury (Qin et al., 2019).

A recent study of *in vitro* and *in vivo* denoted that SIRT1 regulated melatonin’s effects on microglial activation in the context of hypoxia (Merlo et al., 2020). In the study, melatonin significantly attenuated CoCl_2_-induced toxicity in both primary microglia and BV2 mouse microglial cells and indirectly mitigated neuronal damage in neuronal-like cells SH-SY5Y, which were abolished by SIRT1 inhibition. On the other hand, double immunostaining results show that expression of SIRT1 is selectively modified in amoeboid microglia of the corpus callosum with low detectable levels in the hippocampus and cortex in CCAO-induced hypoxic rats. Melatonin reversed the effect of hypoxia on decreased nuclear localization of SIRT1 in hypoxic animals. Moreover, recent study found that autophagy could stimulate the polarization of microglia toward the anti-inflammatory M2 phenotype and reduce inflammatory injury (Zhuang et al., 2020). A study of an MCAO model demonstrated that DJ-1 regulates the polarization of microglia in ischemic rats *via* measuring the expression of microglial polarization markers, which was abolished by the SIRT1 inhibitor administration. And they further investigated the specific mechanism by which DJ-1 affects polarization *via* SIRT1. It was found that DJ-1 possibly activates the Atg5-Atg12-Atg16L1 complex in a SIRT1-dependent fashion, thus promoting autophagic activity and polarization of microglia (Zhao et al., 2022).

### Endothelial cells

Endothelial cells (ECs) are major cellular elements of the blood-brain barrier (BBB) and play a significant role in BBB function, normal physiological brain function and connections between neurons and glial cells. During the process of cerebral ischemia, dysfunction or death of ECs was induced by multiple cell death pathways, such as necroptosis, apoptosis, ferroptosis, and autophagy, leading to increased vascular permeability, BBB disruption and brain edema (Tuo et al., 2022).

Recently, growing data suggests that the neuroprotection of SIRT1 is related to the amelioration of brain endothelial cell injury. An *in vitro* study of brain microvascular endothelial cells (BMECs) model of OGD/R showed that activation of the SIRT1/FOXO3a/NF-κB pathways by donepezil could improve the cell migration, cell viability and angiogenesis, alleviate OGD/R-stimulated cell permeability and increase tight junction protein expression in human BMECs, while the above protective effects were reversed by SIRT1 inhibition (Sun and Liu, 2022). Similarly, another study of hypoxia and high glucose-induced model demonstrated that the SIRT1/HIF-1α/VEGF pathway activated by dipeptidyl peptidase 4 (DPP-4) inhibitor linagliptin enhanced migration and proliferation and exert neuroprotective effects in rat BMECs (Mi et al., 2019). Moreover, a study of OGD/R revealed that the serine/threonine-specific protein kinase (CaMKK) alleviated ischemic brain damage by protecting the microvascular system and decreasing the infiltration of proinflammatory factors in a SIRT1-dependent manner after stroke (Sun et al., 2019a). Intriguingly, recent research has indicated that there exist interactions between SIRT1 and autophagy in brain endothelial cells under oxygen-glucose deprivation.

In an *in vitro* study of OGD/R, the MALAT1 (a long noncoding RNA) and LC3BII levels were increased and p62 levels were reduced after OGD-induced injury, while inhibition of MALAT1 reduced the activation of autophagy and cell survival. The study further demonstrated that MALAT1 suppresses miR-200c-3p expression *via* directly binding to miR-200c-3p. Additionally, miR-200c-3p reduced autophagy and cell survival in BMECs by attaching to SIRT1’s 3′UTR, while MALAT1 reversed this inhibitory effect of miR-200c-3p. These findings revealed a novel Malat1-miR-200c-3p-SIRT1 pathway in the modulation of autophagy in OGD/R-induced brain endothelial cells injury. Similarly, in a study of OGD/R stimulated human umbilical vein endothelial cells (HUVECs), Protocatechualdehyde (PCA) inhibited HUVECs apoptosis-related markers (cleaved-caspase3 and Bax), induced autophagy-related protein P62 expression and elevated the expression of SIRT1, while the anti-apoptotic and pro-autophagy abilities of PCA were abolished by SIRT1 inhibition (Cao et al., 2022). The above studies denoted a novel relationship between SIRT1 and autophagy in brain endothelial cells. But there is a lack of relevant research on the dynamic interactions between different cell types within the brain parenchyma and the neurovascular unit under cerebral I/R conditions, it needs to be further explored.

## Conclusion and future prospects

**FIGURE 1:**
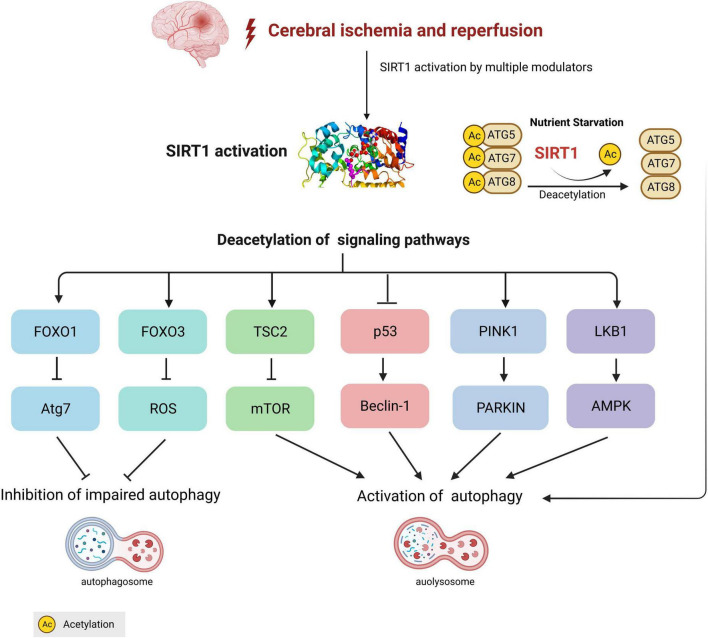
Crosstalk between silent information regulation protein 1 (SIRT1) and autophagy in cerebral ischemia/reperfusion. During the process of cerebral ischemia, SIRT1-mediated different autophagy signaling pathways are involved in the activation or inhibition of autophagy. Under nutrient starvation conditions, SIRT1 directly deacetylates autophagy-related components, including Atg5, Atg7, and Atg8 (LC3) to form ATG 5-7-8 complex and activates autophagy.

Taken together, accumulating evidence has favored a unique role for autophagy and SIRT1-mediated autophagy in the pathogenesis and management of cerebral ischemia and I/R injury (Figure 1). Although autophagy has no unified role in cerebral I/R injury, with substantial progress being achieved on the functions of autophagy, it is generally believed that it exerts neuroprotective effects at least to a certain extent. Moreover, as the crosstalk between SIRT1 and autophagy in cerebral ischemia has been increasingly highlighted, clinical and experimental research has been performed to validate the administration of SIRT1 modulators as a potential therapeutic strategy for cerebral I/R injury by targeting the SIRT1-autophagy axis (Table 2).

**TABLE 2:** Mechanisms of therapeutic compounds on silent information regulator 1 (SIRT1)-mediated autophagy in different ischemic models.

Compounds	Name/Source	Models	Mechanism	References
Resveratrol	A natural polyphenol	Model of subarachnoid hemorrhage	Resveratrol diminished the activation of microglia and the release of inflammatory cytokines by the AMPK/SIRT1-mediated autophagy.	Li and Han, 2018
		MCAO	Resveratrol increased the phosphorylation of AMPK in the cerebral cortex and decreased the infarct area in cerebral I/R rats through promoting mitophagy.	Pineda-Ramírez et al., 2020
		MCAO	Resveratrol attenuated cerebral infarct volume and neuronal apoptosis in MCAO rats *via* the PI3K/AKT/mTOR pathway	Hou et al., 2018
Apelin-36	An endogenous peptide	OGD/R	Apelin-36 reduces mitochondrial apoptosis and improves cell viability in HT22 cells by activating SIRT1, subsequently promoting PINK1 expression levels consistent with the translocation of Parkin to mitochondria.	Shao et al., 2021
CERKL	Ceramide Kinase-Like Protein	OGD/R	CERKL alleviated neuronal damage through activating mitophagy in a SIRT1/PINK1/Parkin-dependent pathway in human neuroblastoma cells.	Huang et al., 2022
NMNAT1	Nicotinamide mononucleotide adenylyltransferase 1	MCAO	NMNAT1 protects against acute ischemic stroke in aged rats by inducing autophagy *via* regulating the SIRT1/mTOR pathway.	Wang et al., 2019
Nampt	Nicotinamide phosphoribosyl transferase	MCAO OGD	Nampt promotes neuronal survival through inducing autophagy *via* modulating the TSC2-mTOR-S6K1 signaling pathway in a SIRT1-dependent manner both *in vivo* and *in vitro*.	Wang et al., 2012
Melatonin	A human hormone	Neonatal model of hypoxia-ischemia	Melatonin reduces activation of the early stages of intrinsic apoptosis, outer mitochondrial membrane permeabilization and glial cell activation through the SIRT1-p53-mediated signaling autophagy pathway.	Carloni et al., 2017
Simvastatin preconditioning	An oral antilipemic agent	Neonatal model of hypoxia-ischemia	The mechanism is mediated by the preservation of SIRT1, thus suppressing p53 expression and acetylation levels and conferring neuroprotection against hypoxia-ischemia-induced brain damage in neonatal rats.	Carloni and Balduini, 2020
protocatechualdehyde	A major bioactive component of the traditional Chinese medicine *Salvia miltiorrhiza* Bunge (Lamiaceae)	OGD/R	The mechanisms involve upregulation of SIRT1 and inhibition of P53 and cell apoptosis in OGD/R-induced human umbilical vein endothelial cells (HUVECs), as well as activation of autophagy (increased expression of autophagy markers P62, LC3 II/I, and Beclin-1).	Cao et al., 2022

AMPK, adenosine monophosphate (AMP)-activated kinase; I/R, ischemia/reperfusion; MCAO, middle cerebral artery occlusion; OGD/R, oxygen-glucose deprivation/reoxygenation; PI3K, phosphoinositide 3-kinase; mTOR, mammalian target of rapamycin; FOXO, forkhead box protein O; PINK, PTEN-induced putative kinase 1; CERKL, Ceramide Kinase-like Protein; PGC-1α, peroxisome proliferator-activated receptor-gamma coactivator; TSC2, tuberous sclerosis complex-2; NMNAT1, Nicotinamide mononucleotide adenylyltransferase 1.

Mounting evidence revealed that resveratrol has various therapeutic effects, such as anti-apoptotic, anti-oxidative, anti-inflammatory, and neuroprotective functions. A recent clinical study revealed that long-term resveratrol supplementation had positive effects and served as a secondary prophylaxis for patients with stroke. Stoke patients who took oral resveratrol supplements (100 or 200 mg, respectively) daily for 12 months exhibited a significant reduction in mean body mass index (BMI), blood glucose levels, cholesterol, and LDL cholesterol (Fodor et al., 2018). Although the mechanism underlying the effect of resveratrol on stroke patients remains unclear, several aforementioned experimental studies have demonstrated that resveratrol may target the SIRT1-autophagy axis to provide neuroprotective effects (He et al., 2017b; Hou et al., 2018; Li et al., 2021). Therefore, the clinical and experimental studies both suggest resveratrol as a possible treatment for cerebral ischemia that targets the SIRT1-mediated autophagic pathway.

Apart from resveratrol, some other findings showed that pharmacological compounds could exert neuroprotection through SIRT1-mediated autophagy, such as simvastatin and protocatechualdehyde. A multicenter clinical trial revealed that simvastatin treatment combined with thrombolysis is safe and efficacious in the acute phase of ischemic stroke. Patients with acute ischemic stroke who received oral simvastatin 40 mg per day for 90 days demonstrated safe and low rates of bleeding events compared with the placebo patients (Montaner et al., 2016). An animal study revealed that simvastatin preconditioning provides beneficial effects against hypoxia-ischemia in neonatal rats *via* targeting SIRT1-mediated autophagy (Carloni and Balduini, 2020). This study presents further preclinical evidence of the beneficial effects of statins in brain tissue, confirming their role in improving stroke outcomes after preventative therapies.

However, some questions remain unanswered. For example, how can we precisely monitor autophagy during the cerebral ischemia process? What functions does autophagy serve in the various phases or types of cerebral I/R injury? Furthermore, the biological functions of SIRT1 are complex and wide. The precise molecular mechanisms and negative effects of SIRT1’s autophagic regulation in cerebral I/R injury remains to be explored. Moreover, the evidence of neuroprotective effects of SIRT1-mediated autophagy from clinical studies is not enough and needed to be further elucidated. Continued discovery and in-depth understanding of crosstalk mechanisms of SIRT1 and autophagy will provide new strategies and treatments for cerebral I/R injury.

## Author contributions

YT, JX, and XC reviewed the literature and drafted the manuscript. XC and LS made the tables and figures. LX and XC finalized the manuscript and provided suggestions to improve it. All authors participated in designing the concept of this manuscript.
